# Boosting In-Vivo Anti-Tumor Immunity with an Oral Microparticulate Breast Cancer Vaccine and Low-Dose Cyclophosphamide

**DOI:** 10.3390/vaccines11030543

**Published:** 2023-02-24

**Authors:** Nihal Mulla, Lipika Chablani, Ashwin C. Parenky, Martin J. D’Souza

**Affiliations:** 1 Department of Pharmaceutical & Administrative Sciences, College of Pharmacy & Health Sciences, Western New England University, Springfield, MA 01119, USA; 2 Department of Pharmaceutical Sciences, Wegmans School of Pharmacy, St. John Fisher University, Rochester, NY 14618, USA; 3 Vaccine Nanotechnology Laboratory, Department of Pharmaceutical Sciences, College of Pharmacy and Health Sciences, Mercer University, Atlanta, GA 30341, USA

**Keywords:** microparticles, cancer vaccines, breast cancer, regulatory T cells, cyclophosphamide

## Abstract

Tumor cells express antigens that should induce immune-mediated rejection; however, spontaneous rejection of established tumors is rare. Recent evidence suggests that patients suffering from cancer exhibit an elevation in regulatory T cells population, a subset of CD4+ T cells, which suppress tumor recognition and elimination by cytotoxic T cells. This study investigates immunotherapeutic strategies to overcome the immunosuppressive effects exerted by regulatory T cells. A novel immunotherapeutic strategy was developed by simultaneous administration of oral microparticulate breast cancer vaccines and cyclophosphamide, a regulatory T cell inhibitor. Breast cancer vaccine microparticles were prepared by spray drying, and administered orally to female mice inoculated with 4TO7 murine breast cancer cells in combination with a low dose of intraperitoneally administered cyclophosphamide. Mice receiving the combination of vaccine microparticles and cyclophosphamide exhibited maximal tumor regression and the highest survival rate compared with the control groups. This study highlights the importance of cancer vaccination along with regulatory T cell depletion in cancer therapy, and suggests that a low dose of cyclophosphamide that specifically and significantly depletes regulatory T cells may be a highly effective immunotherapeutic strategy for the treatment of cancer.

## 1. Introduction

The American Cancer Society predicts approximately 287,850 new cases of breast cancer in the USA by the end of 2022. According to the National Cancer Institute, around 51,400 deaths are projected to occur due to breast cancer in the USA in the same year [1]. Currently, patients with breast cancer are treated by surgical removal of tumor cells followed by chemotherapy, radiation therapy, or hormone therapy. Both chemotherapy and radiation therapy do not act specifically against tumor cells and are therefore associated with serious side effects against normal cells [2]. As a result of these side effects, both therapies require regulation of doses and exposure times. Moreover, tumors are highly adaptive and possess the ability to become insensitive towards chemotherapy and radiation therapy [2]. Even with the advancements in therapeutic strategies against cancer, large numbers of patients face the risk of relapse [3]. The aim of effective cancer therapy is to inhibit micrometastatic disease, avoid recurrence, and enhance long-term overall survival.

Immunotherapy is a treatment strategy that utilizes the immune system to fight cancer. Treatment of cancer by immunotherapy involves two steps: (a) stimulation of immune cells against cancer cells, and (b) depletion of immunosuppressive regulatory T cells. Cancer cells overexpress specific immunogenic proteins, thus representing a potential source of antigens for immunotherapy [4]. Previously, we explored tumor-associated antigens (TAAs) as immunogens for generating an immune response against cancer cells [5,6,7,8,9,10]. Although these TAAs have been reported to elicit weak immune responses, their immunogenicity may be enhanced by using adjuvants or chemotherapeutic drugs [6,11,12]. A recently developed cell-based form of immunotherapy against prostate cancer (Provenge^®^) involves isolating dendritic cells (DCs) from patients with prostate cancer and stimulating them with a prostate-specific fusion protein, followed by the reintroduction of these DCs into the patient. In clinical trials, this procedure, when carried out three times, was found to marginally increase survival in prostate cancer patients by four months [13]. The cancer vaccine developed in our laboratory consists of a pool of TAAs prepared by lysing tumor cells [7,10,14,15]. Vaccine microparticles were prepared by spray drying these TAAs with a blend of enteric coating polymers. By using TAAs as the cancer vaccine, we ensured that an immune response would be generated against all proteins overexpressed by the tumor cells. We evaluated the efficacy of orally administered cancer vaccines in breast, skin, ovarian, and prostate cancer models. Our findings indicated that oral vaccination induced a robust immune response against the respective TAAs in each cancer model. In order to evaluate the efficacy of the vaccine, we challenged the immunized mice with live tumor cells. Tumor growth was suppressed in animals receiving cancer vaccine microparticles. In order to elucidate the immune response generated against the TAAs following the challenge, we quantified the population of CD8+ and CD4+ T cells in immune organs such as the spleen and lymph nodes. Tumor growth was suppressed in vaccinated animals, demonstrating that vaccine microparticles induced the development of adaptive immunity against the tumor antigens. Vaccinated animals showed significantly higher populations of cytotoxic T cells and T helper cells compared with unvaccinated animals [5,7,10,16].

Polymeric vaccine microparticles offer several advantages, such as protection from gastric pH conditions, sustained release, and higher vaccine uptake by phagocytic cells [5,7,17,18,19,20]. A microfold cell (M cell) targeting ligand, Aleuria aurantia lectin (AAL), was incorporated into the vaccine formulation. M cells, found in the follicle-associated epithelium of Peyer’s patches, transport vaccine particles from the gut lumen to immune cells across the epithelial barrier [21]. Vaccine delivery by microparticles leads to better uptake by antigen-presenting cells (APCs) [22]. DCs, a type of APCs, are one of the major effector cells of the immune system. These cells form an important part of the linkage between the innate and adaptive immune response. DCs engulf and lyse the microparticles to express the antigens or their fragments on their surfaces as part of the MHC I or MHC II complex [18,23,24,25]. In this study, we evaluate the effects of antigen delivery via microparticles on the expression of various cell-surface antigen-presenting and co-stimulatory molecules, such as CD40, CD86, MHC I, and MHC II.

This study also evaluates the benefits of combining oral vaccination with an immunomodulatory drug. The success of vaccine-based therapy lies in its ability to induce a strong and specific immune response against the antigen of interest. The roles of CD8+ and CD4+ T cells in cancer vaccine therapy are well established. Regulatory T cells, known to suppress antigen-specific T-cell responses, are found in higher numbers in patients with cancer [26,27,28]. The presence of immunosuppressive regulatory T cells limits the effectiveness of cancer vaccines [26,27]. Therefore, our study aimed to deplete regulatory T cells and simultaneously induce CD8+ and CD4+ T cell immune responses against TAAs. Cyclophosphamide is one of the most widely used immunomodulatory drugs for inhibiting regulatory T cells [29,30,31]. The survival benefits associated with treatment using single therapeutic agents are limited; therefore, we evaluated the advantages of combinatorial therapy involving the administration of vaccine microparticles and immunomodulatory drugs. Our findings indicate that vaccine microparticles induce significantly higher levels of DC activation and antigen presentation than vaccine solution. Furthermore, we tested the effects of combining oral vaccines and intraperitoneally administered (i.p.) cyclophosphamide on breast cancer development in a mouse model.

## 2. Materials and Methods

### 2.1. Materials

Dulbecco’s Modified Eagle medium (DMEM), Roswell Park Memorial Institute (RPMI) medium, and Dulbecco’s phosphate buffered saline (DPBS) were purchased from Atlanta Biologicals, Lawrenceville, GA, USA. Hydroxyl propyl methyl cellulose acetate succinate (HPMCAS), ethyl cellulose (EC) and cellulose acetate phthalate (CPD) were purchased from AQUACOAT, FMC Biopolymers. Aleuria aurantia lectin (AAL) was obtained from Vector Labs Inc., Newark, CA, USA. Cyclophosphamide, bovine albumin-fluorescein isothiocyanate (FITC–albumin), and trehalose were obtained from Sigma Aldrich, St. Louis, MO, USA. Flow cytometer markers, such as anti-CD4, anti-CD8a, anti-CD62L, anti-CD40, anti-CD80, anti-mouse Foxp3 and anti-MHC-II antibodies, were bought from eBioscience, San Diego, CA, USA.

### 2.2. Mice and Cell Lines

Six-to-eight-week-old female BALB/c mice were purchased from Charles River Laboratories, MA, USA. The mice were housed according to Mercer University’s Institutional Animal Care and Use Committee (IACUC) guidelines. The murine breast cancer cell line (4TO7) was a gift from Dr. Fred Miller, Barbara Ann Karmanos Cancer Institute, Detroit, MI, USA. The DCs (DC 2.4) were a kind gift from Dr. Kenneth L. Rock (Dana-Farber Cancer Institute, Inc., Boston, MA, USA)

### 2.3. Tumor-Associated Antigens

The TAAs were extracted from the murine breast cancer cell line (4TO7) using hypotonic lysis buffer (10 mM Tris and 10 mM NaCl) and further subjected to five freeze/thaw cycles at −80 °C and 37 °C for 10 min each. At the end of the final freeze/thaw cycle, cell lysis was confirmed using trypan blue dye exclusion assay. The presence of dead cells confirmed the end point of freeze/thaw cycles. The whole cell lysate (WCL) thus obtained was stored at −80 °C for subsequent extraction of TAAs.

### 2.4. Protein Quantification of Tumor-Associated Antigens

The WCL was characterized for protein content using Bio-Rad DC total protein assay. Standard protein bovine serum albumin (BSA) was used to prepare the standard curve (1–0.062 mg/mL), which was used to estimate the protein concentration of TAAs in WCL.

### 2.5. Preparation of Vaccine Microparticles

The 4T07 antigen-loaded vaccine particles were prepared using a previously reported method [7,32]. Microparticles were optimized for particle size, zeta potential, and sustained vaccine release [32]. Briefly, vaccine microparticles were prepared by spray drying an aqueous suspension containing TAAs, EC, trehalose, HPMCAS, and M-cell targeting agent AAL. Vaccine microparticle formulation was prepared by using EC:HPMCAS in an 8:1 ratio. HPMCAS was dissolved in deionized water (DI) by adjusting the pH to 8 by adding 0.1 M NAOH and stirring overnight. From an EC stock suspension (30% *w*/*v*), equivalent suspension containing 8 times the concentration of HPMCAS solid content was added to the HPMCAS solution. TAAs equivalent to 10% *w*/*w* concentration were added to the above solution along with 5% *w*/*v* trehalose and 0.01% *w*/*v* Tween 20. AAL (0.25% *w*/*w*) was added to the formulation to target the particle to M cells in the Peyer’s patches of the small intestine. This final matrix was spray dried using a Buchi 290 Mini Spray Dryer (Buchi Corporation, Newcastle, DE, USA) with an inlet temperature of 125 °C and outlet temperature of 80 °C. The particles were stored at −20 °C until further use.

### 2.6. Characterization of Size, Shape, and Charge of Microparticles

Spray-dried particles were analyzed for their size, morphology, and zeta potential. Antigen-loaded microparticles (10 mg) were suspended in 1 mL of citrate buffer (100 mM, pH 4.0) and particle size was measured using a Spectrex laser particle counter (Spectrex Corp, Redwood City, CA, USA). The zeta potential of the microparticles was measured using a Malvern Zetasizer Nano ZS (Malvern Instruments, Worcestershire, UK). For morphology studies, vaccine microparticles were visualized using a scanning electron microscope (Phenom World Pure Scanning electron microscope, Phoenix, AZ, USA).

### 2.7. Entrapment Efficiency

Total antigen entrapment efficiency was evaluated by dissolving 5 mg of vaccine particles in 1 mL of DPBS. The total antigen content of this solution was analyzed using Bio-Rad DC total protein assay. A standard curve was prepared using BSA to calculate the antigen content in particles.

### 2.8. In Vitro Antigen Release from Microparticles

In order to elucidate the mechanism by which antigens are released from the microparticles, 10 mg of microparticles were exposed to 1 mL release media under murine stomach pH (pH 3) conditions for 30 min, and then to intestinal pH (pH 5). A 100-µL sample was taken at 0.5, 1, 2, 3, 4, 5, and 6 h. An appropriate volume of buffer was replaced to maintain sink conditions. The concentration of antigen protein in the release media was evaluated using Biorad DC total protein assay.

### 2.9. Microparticle Uptake by Dendritic Cells

Hardy et al. reported that administration in nano/microparticulate formulation results in the delivery of higher amounts of antigen [18]. In order to evaluate the uptake of microparticles by DCs, we prepared microparticles containing 1% *w*/*w* fluorescein isothiocyanate (FITC) conjugated to BSA, using the method previously described. The uptake of BSA-FITC microparticles by DCs was compared with that of BSA-FITC solution. Ten thousand DCs (DC 2.4) were seeded onto a 96 well plate and 100 µg of particles were added to each well (*n* = 3), with 100 µL of 1% *w*/*v* FITC-BSA solution added to control wells (*n* = 3). DCs were incubated with microparticles for 2 h at 37 °C. After incubation, cells were washed with PBS to remove free FITC-BSA, collected, and analyzed for fluorescence using the BD Accuri C6 flow cytometer.

### 2.10. Dendritic Cell Activation and Antigen Presentation

A large number of activated immune cells, such as DCs and macrophages, release nitric oxide on exposure to antigens [33]. Nitric oxide regulates the functional activity, growth, and death of numerous immune and inflammatory cell types, including macrophages, T lymphocytes, DCs, mast cells, neutrophils, and natural killer (NK) cells. We evaluated the amount of nitric oxide released by DCs after being pulsed with vaccine microparticles. DCS (DC 2.4) (300,000/well) were pulsed with vaccine particles (300 µg) and blank microparticles (without vaccine) in a 48-well plate for 16 h. Antigen (cell lysate) solution (30 µL of 1 mg/mL stock) was added to control wells (*n* = 3). After 16 h, the supernatant was harvested and analyzed for nitric oxide concentration using the Greiss chemical method. The Greiss reagent was prepared by mixing equal volumes of 1% sulfanilamide and 0.1% N-(1-napthylethyldiamine) solutions. One hundred microliters of supernatant were transferred to a 96-well plate to which 100 µL of Greiss reagent were added. The plate was incubated for 10 min and read at a wavelength of 540 nm using a microplate reader (EL312e; BIO-TEK Instruments, Winooski, VT, USA). The first step converts the nitrates to nitrite and the second step uses Griess reagent to convert nitrite to a deep purple azo compound. The purple-colored azo compound absorbs at 540 nm. The nitrite concentration was calculated using the standard curve of NaNO_2_ (1 mM stock concentration in distilled water further diluted to the highest standard at 100 mM, followed by serial dilutions to 1.56 mM). The cells were collected and analyzed for cell-surface expression markers, and incubated with fluorescently tagged antibodies. Post-incubation, cells were washed with Hanks Balanced Salt Solution (HBSS), and unbound antibody was removed by centrifugation at 1500 rpm for 10 min. Subsequently, the cells were analyzed by flow cytometry (Becton Dickinson) for expression of the following co-stimulatory molecules (i) CD86, (ii) CD40, (iii) MHC I, and (iv) MHC II.

### 2.11. Anti-Tumor Efficacy of Orally Administered Breast Cancer Vaccine Microparticles

Female BALB/c mice were subcutaneously injected with 100 µL of cell suspension containing 1 × 10^6^ 4TO7 murine breast cancer cells. The day of tumor inoculation into the mice was designated day 0. Vaccine microparticles (10 mg) were administered orally four days post-tumor inoculation. A low dose of cyclophosphamide (50 mg/kg) was administered via the intraperitoneal route three days prior to the next vaccine dose. Animals received four doses of vaccine microparticles with a 5-day interval between each dose.

Four doses of microparticles (10 mg/dose), with or without the vaccine, were administered orally on day three following each cyclophosphamide dose. In order to elucidate the effects of cyclophosphamide on the immune response, mice were dosed, as shown in Table 1. Tumor volumes were measured for four weeks post-tumor inoculation. Animals were then euthanized as per Mercer University IACUC protocols. Tumor volume was measured for 52 days using the following formula:

Tumor volume = 1/2 [length × (width)^2^]

Mice were observed for 52 days to evaluate the vaccine’s induction of long-term immunity. We hypothesized that the animals receiving a combination of vaccine and cyclophosphamide would exhibit resistance to cancer recurrence for a longer duration compared with unvaccinated animals or animals receiving only cyclophosphamide. Animals were euthanized according to Mercer University IACUC protocols if any discomfort was observed.

### 2.12. Determination of T Cell-Based Cellular Response

Spleens were collected and processed to obtain a single cell suspension and analyzed by flow cytometry using BD accuri^®^ C6 flow cytometer. Briefly, abdominal cavities were excised, and spleens were collected under aseptic conditions. A single suspension of spleen cells was prepared by passing through a fine mesh. Erythrocytes were lysed using ammonium chloride and potassium bicarbonate lysis buffer. Cells were then washed three times using Hank’s balanced salt solution and labeled with anti-mouse CD8a FITC (for cytotoxic T cells), anti-mouse CD 4 PE (for CD + helper T cells), anti-mouse CD4/CD25 PE (for regulatory T cells), and anti-mouse CD62L FITC (for T memory cells).

### 2.13. Survival Curve and Statistical Analysis

Survival curves for tumor-free survival were plotted according to the Kaplan-Meier method. The data were analyzed using GraphPad Prism (GraphPad software, La Jolla, CA, USA). Statistical significance was determined using a one-way ANOVA analysis of data from three independent experiments. Values of *p* < 0.05 were considered statistically significant.

## 3. Results

### 3.1. Quantification of TAAs

The protein content of TAAs was measured using the Bio-Rad DC total protein assay kit. The total protein content of TAAs prepared from 5 × 10^6^ cells was 2–3 mg/mL. TAAs were used as the vaccine component for the formulation of vaccine-loaded microparticles.

### 3.2. Characterization of Size, Shape, and Charge of Microparticles

**Figure 1:**
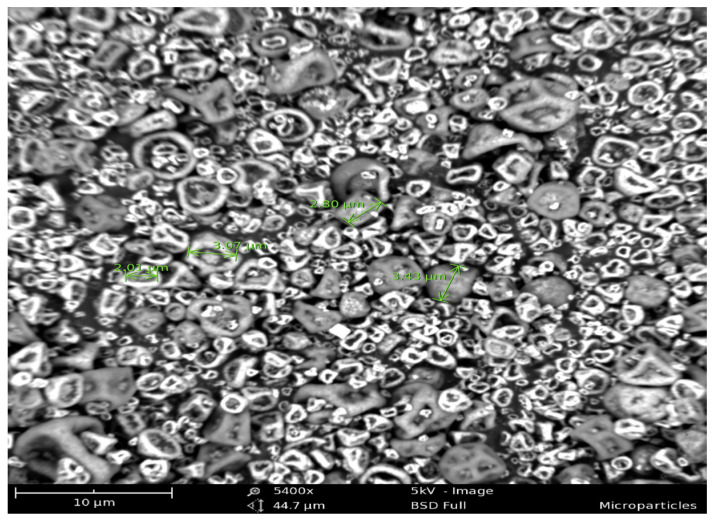
Scanning electron micrograph of polymeric microparticles. SEM images were captured using Phenom Desktop SEM^®^ by placing the microparticles on a conductive carbon tape and observed at 4100 × 5 kV. Microparticles exhibited an irregular shape with a particle size range of 1 to 4 µm.

The particles, whose shape and size were visualized using scanning electron microscopy (Figure 1), ranged between 1–4 µm in size and exhibited an irregular shape with a positive zeta potential of around 7 ± 1.5 mV.

### 3.3. Entrapment Efficiency

The protein content of the particles was determined by dissolving them in DPBS at pH 7.4. The released protein was collected by centrifugation, and the supernatant was analyzed using the Bio-rad DC total protein assay. The entrapment efficiency of vaccine microparticles, which was used to calculate the amount of vaccine microparticles required for the in vivo efficacy study, was 80 ± 3.5%.

### 3.4. In Vitro Antigen Release from Microparticles

**Figure 2:**
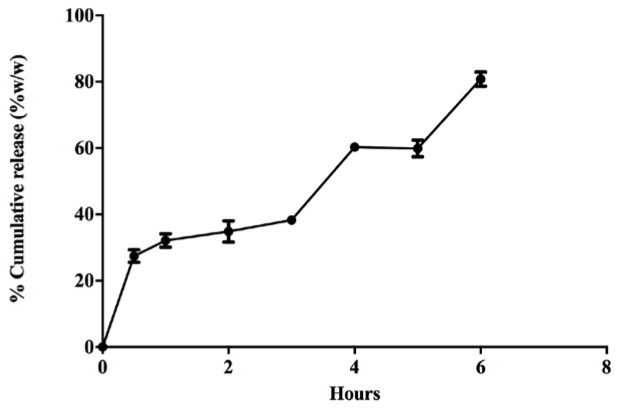
Vaccine release was evaluated under stomach pH conditions (pH 3) for 30 min, followed by intestinal pH conditions (pH 5) to estimate the amount of protein released from 10 mg of microparticles after oral dosing. Protein concentration was evaluated using the Biorad DC total protein assay method. About 70% of the vaccine remained encapsulated under stomach pH conditions. The microparticulate formulation resulted in the sustained release of the vaccine over a period of 6 h.

The microparticles, prepared using enteric coating polymers such as EC and HPMCAS, released about 30% w/w of antigen at stomach pH (pH 3). The initial burst release was observed due to the release of protein adsorbed on the surface of the particles. Therefore, about 70% of the antigen still present in the matrix was eventually taken up by M cells in the Peyer’s patches of the small intestine (Figure 2). Protein and enzyme-free dissolution medium were used to minimize interaction with the antigen release profile.

### 3.5. Microparticle Internalization by Dendritic Cells

**Figure 3:**
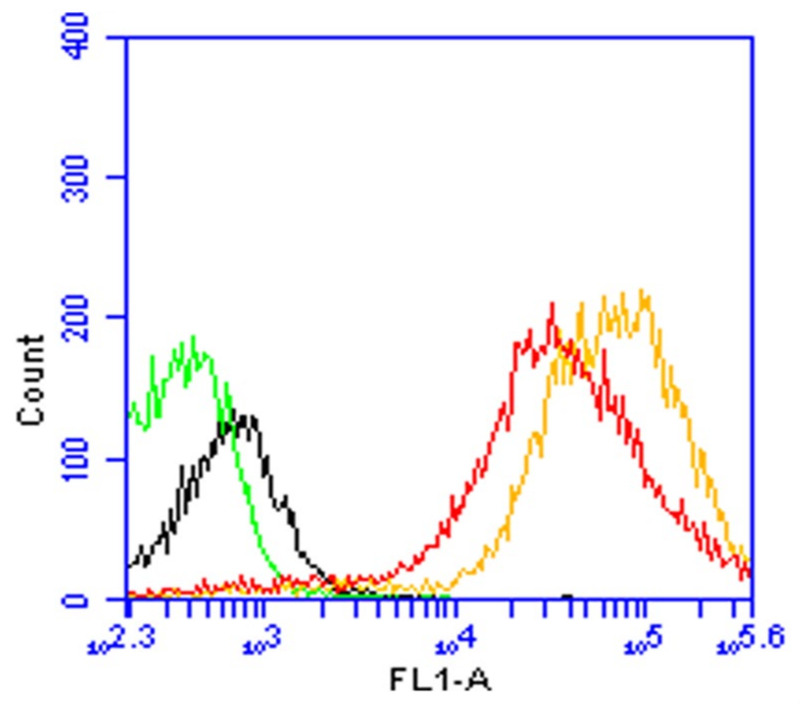
Flow cytometric analysis of the uptake of 100 µg of FITC-BSA-loaded microparticles (yellow curve) and FITC-BSA solution (red curve) by DCs (DC 2.4) after 2 h of incubation at 37 °C. The uptake of FITC-BSA loaded microparticles was significantly higher than that of the FITC-BSA solution (*p* < 0.05). The green and the black curves represent cells that did not receive any fluorescent dye.

DCs (DC 2.4) were exposed to FITC-labeled BSA-loaded particles and the corresponding control solution. Cells exposed to microparticles containing FITC-labeled BSA-loaded particles exhibited three-fold higher mean fluorescence intensity than cells exposed to FITC-labeled BSA solution (Figure 3), thus confirming that the microparticulate formulation enables the delivery of a higher amount of antigen to DCs than the solution form. The mechanism of internalization of particulate antigens plays an important role in determining the specificity of the immune response.

### 3.6. Nitric Oxide Assay

**Figure 4:**
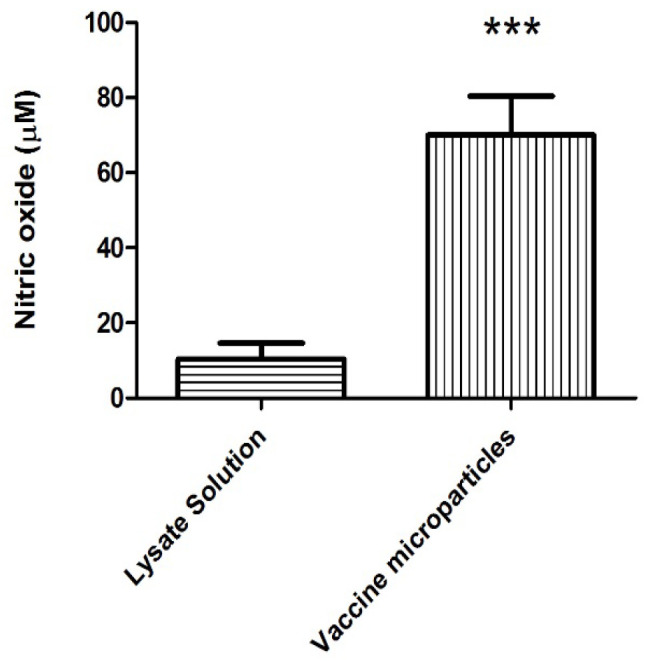
Nitric oxide released by 300,000 DCs (DC 2.4) following incubation with lysate (vaccine) solution and 300 µg of vaccine-loaded microparticles. Vaccine-loaded microparticles induced a higher nitric oxide release compared with lysate solution. (*** *p* < 0.001).

Nitric oxide is an important marker of the innate immune response. APCs, such as DCs, release nitric oxide on exposure to antigen. Our findings indicated that the vaccine microparticles induced the DCs to release a significantly higher amount of nitric oxide than the solution counterpart (70.03 ± 10.32 µM compared with 10.37 ± 4.21 µM of nitrite, respectively) (Figure 4). The nitric oxide basal levels of cells alone were subtracted from both the treatment groups.

### 3.7. Vaccine Microparticles Induce Dendritic Cell Activation and Antigen Presentation

**Figure 5:**
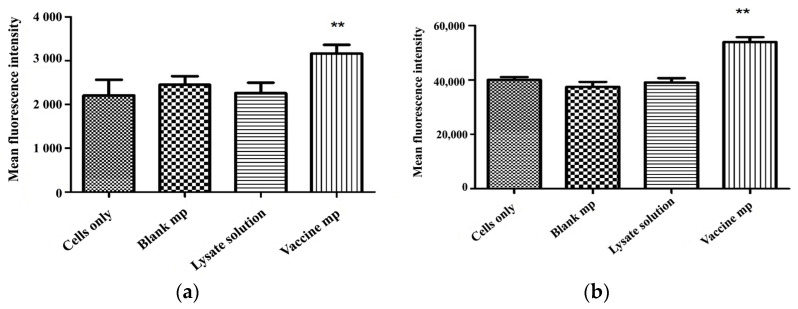

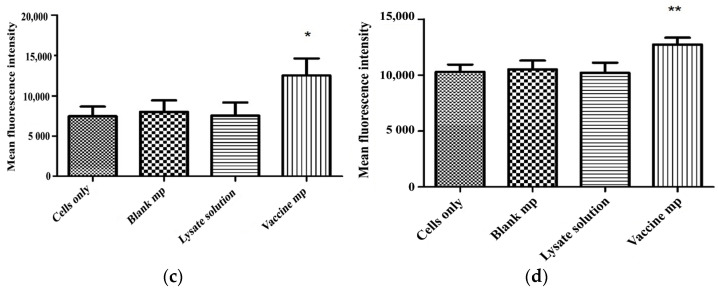
Analysis of the effects of microparticles on the following co-stimulatory molecules in DCs: (**a**) CD40, (**b**) CD86, (**c**) MHC I, (**d**) and MHC II. DCs (DC 2.4) (300,000 cells) were incubated with 300 µg of vaccine microparticles for 16 h. Whole cell lysate containing TAAs (30 µL of 1 mg/mL solution) and 300 µg of blaTnk microparticles were used as the control. Vaccine-loaded microparticles induced a significantly higher expression of CD40 (*, *p* < 0.05), CD86, MHC I and MHC II (**, *p* < 0.01) than the control groups.

Dendritic cells were incubated with vaccine microparticles, blank microparticles, and vaccine solution for 16 h. As shown in Figure 5, vaccine microparticles induced the expression of CD40 and CD86, both of which are important for T cell co-stimulation. Vaccine microparticles induced a significantly higher number of CD40 and CD86 cell surface receptors than the vaccine solution and blank microparticles. The surface expression of TAAs as either MHC II or MHC I molecules on APCs depends upon the mechanism of uptake. In this study, antigens were presented as complexes with both MHC II and I molecules.

### 3.8. Combination of Cyclophosphamide and Oral Vaccine Elicits Maximal Tumor Regression

**Figure 6:**
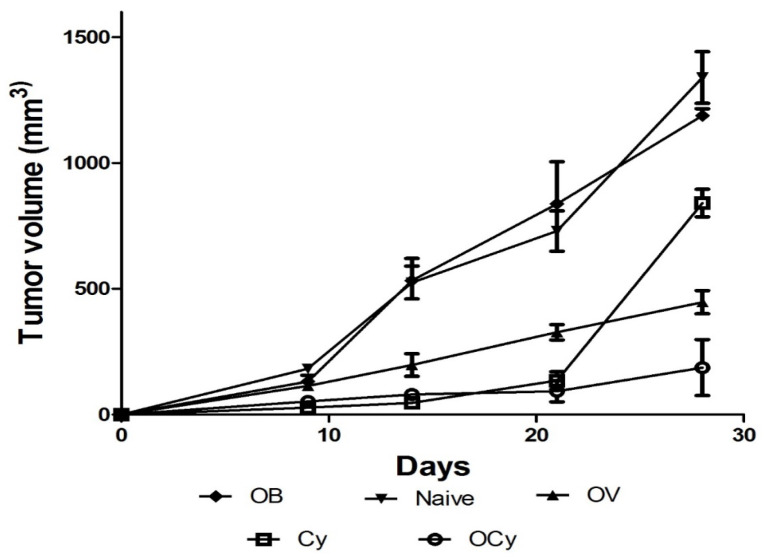
Tumor volumes in animals receiving the following treatments: naïve, blank microparticles (OB), cyclophosphamide (i.p., 50 mg/kg) (Cy), orally administered vaccine microparticles (OV), and combination of vaccine and cyclophosphamide (OCy). The highest inhibition of the growth of breast tumors was achieved by the combination treatment of cyclophosphamide and oral breast cancer vaccine microparticles (*p* < 0.05).

Animals receiving orally administered vaccine microparticles showed significant tumor regression compared with the control groups (Figure 6). The combination of cyclophosphamide and vaccine microparticles caused a significantly higher tumor reduction compared with no (naïve/blank microparticles) or single treatment (*p* < 0.05) at the end of 28 days. Animals receiving orally administered blank microparticles and naïve animals exhibited similar tumor progression, indicating that the microparticles alone did not lead to tumor inhibition.

### 3.9. Kaplan-Meier Survival Curve

**Figure 7:**
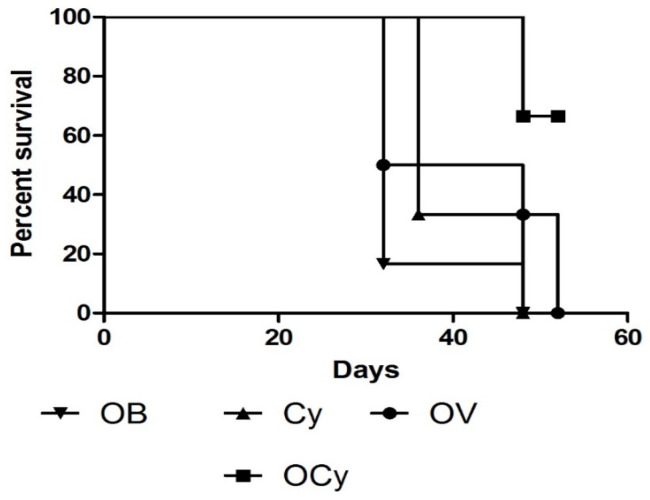
Kaplan–Meier survival curve for animals receiving the following treatments: blank microparticles (OB), cyclophosphamide only (Cy) (i.p., 50 mg/kg), orally administered vaccine microparticles (OV), and a combination of vaccine and cyclophosphamide (OCy). Animals were continuously monitored for 28 days for tumor growth and discomfort. The survival rate was highest in animals receiving a combination of cyclophosphamide and vaccine microparticles.

Mice receiving combination therapy exhibited higher survival rates than the other groups at the end of the 52-day observation period. Naïve animals received no treatment and were euthanized after 28 days (data not shown). Animals that received single or no treatment exhibited significantly higher tumor growth than those receiving combination therapy (Figure 7). The Kaplan–Meier survival curve showed that breast cancer vaccine microparticles induce long-term immunity against cancer cells.

**Figure 8:**
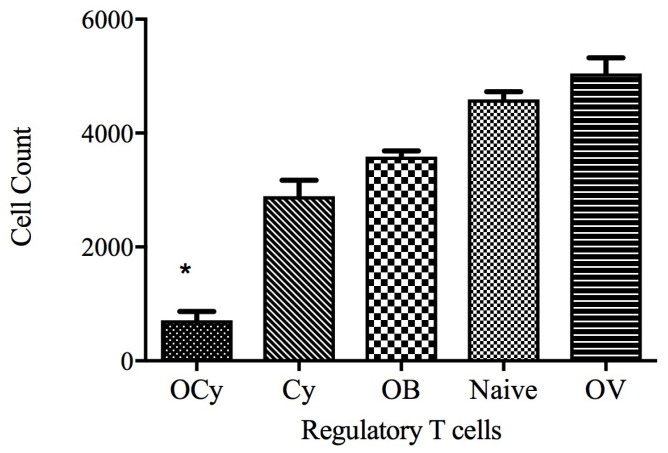
Analysis of the effect of cyclophosphamide (50 mg/kg) on the CD4^+^CD25^+^Foxp3^+^ regulatory T cell population. Cyclophosphamide was administered three days before the booster vaccine doses. Spleens (*n* = 6) were collected, and regulatory T cell populations were analyzed by flow cytometry * *p* < 0.001 (OCy: orally administered vaccine microparticles + cyclophosphamide; OV: orally administered vaccine microparticles; OB: orally administered blank microparticles; Cy: cyclophosphamide).

The tumor volume data were consistent with the findings related to the regulatory T cell and CD 8+ T cell populations. Animals receiving oral vaccine along with low-dose cyclophosphamide intraperitoneally showed a maximal reduction in regulatory T cell levels compared with those receiving no or only cyclophosphamide (Figure 8). The regulatory T cell population depends significantly on the presence and absence of tumors. Animals exhibiting significantly low levels of regulatory T cells showed the highest regression in tumor growth.

**Figure 9:**
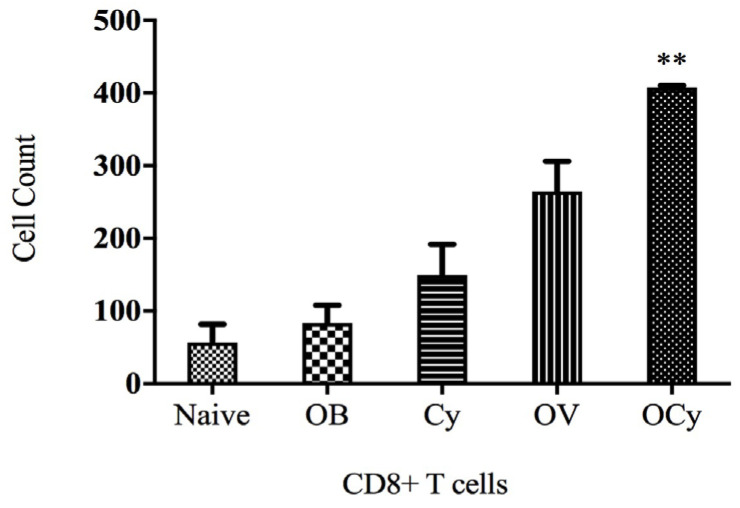
Analysis of the CD8+ T cell population level in the naïve mice and those administered blank microparticles (OB), cyclophosphamide (i.p., 50 mg/kg (Cy)), oral vaccine microparticles (OV), and a combination of vaccine and cyclophosphamide (OCy). The CD8+ T cell population was significantly higher in mice administered low-dose cyclophosphamide and oral vaccination compared with control groups (** *p* < 0.01).

The CD 8+ T cell population, which mounts an immune response against tumor cells, was significantly higher in animals receiving both vaccine and cyclophosphamide (Figure 9). This result was in accordance with the tumor volume data, which indicated that tumor volume was lowest in animals exhibiting the highest CD8+ T cell population. The animals receiving combination therapy (OCy) induced a significantly higher CD8+ T cell population than those receiving the vaccine only (OV).

**Figure 10:**
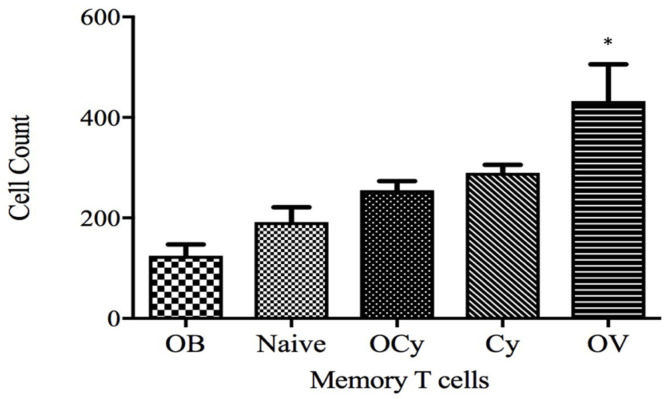
Analysis of the central memory T cell population in naïve mice and those receiving blank microparticles (OB), cyclophosphamide (i.p., 50 mg/kg), oral vaccine microparticles (OV), and a combination of vaccine and cyclophosphamide (OCy). Spleen samples (*n* = 6) were collected 52 days post-inoculation with 4TO7 breast cancer cells. The central memory T cell population was evaluated using fluorescently labeled anti-CD62L antibodies. The animals receiving cyclophosphamide showed significantly lower count of memory T cells (* *p* < 0.01).

In addition to the therapeutic efficacy, the ability of vaccine microparticles to induce central memory T cell response was evaluated. No significant difference in the CD62L + memory T cell population was observed between the treatment groups OCy and Cy (Figure 10). However, the OV group showed significantly higher memory T cell population. Even in low doses, cyclophosphamide could have potentially eradicated memory T cells.

## 4. Discussion

Uninhibited tumor growth occurs due to immune suppression or evasion of immune recognition. The immune system is unable to identify and eliminate tumor cells since the tumor microenvironment consists of specific cytokines that are known to induce the proliferation of immunosuppressive regulatory T cells [34,35]. Therefore, effective tumor immunotherapy must involve the inhibition of regulatory T cells. Several recent studies have shown that a low dose of cyclophosphamide specifically inhibits regulatory T cells [28]. We hypothesized that a combination of cancer vaccine with a low dose of a cyclophosphamide can provide a multitude of benefits, enabling tumors to exhibit higher immunogenicity by eliminating the immunosuppressive environment created by cancer cells.

Our group, and many others, have previously shown that microparticles are taken up by M cells in the small intestine when administered orally [5,7,14,16,36,37,38]. DCs underlying the M cells internalize the vaccine microparticles via phagocytosis or pinocytosis. Subsequently, the vaccine particles, which consist of TAAs, are cleaved into smaller peptides within the intracellular vesicles. Figure 5 shows that, in comparison with antigen-containing solution, microparticulate antigens induce significantly higher antigen presentation and co-stimulatory signals in DCs. We observed a significant increase in cell-surface expression of CD86, which is a co-stimulatory molecule for DC activation. CD86 expression is additionally vital for binding between DCs and T cells. In addition, an increase in the expression of CD40, which is important for binding to the CD40 ligand on T cells, was observed. The mechanism of antigen uptake by DCs affects antigen expression. Multiple studies suggest that soluble antigens are taken up by DCs and degraded slowly in the lysosome, resulting in expression of the antigen in complex with MHC II [39,40]. As reported by Hanlon et al., the uptake of vaccine solution is a slow and inefficient process that may not induce a strong CD8+ T cell immune response [40,41]. However, particulate antigens are taken up more efficiently by cells than the solution and expressed in complex with MHC I molecules. Proteasomes produce peptide fragments from all proteins present in the cell including self and non-self-proteins. MHC I is synthesized by ribosomes on the endoplasmic reticulum. Therefore, when a microparticle containing an antigen or protein is taken up, part of the antigen is released into the cytosol and transported by the transporter-associated protein (TAP) into the endoplasmic reticulum and presented as an MHC I complex [41]. The incubation of vaccine microparticles with DCs improved antigen presentation as both MHC I and MHC II complexes compared with the vaccine solution (Figure 5). These findings confirm that DCs take up vaccine microparticles more efficiently than soluble antigen, which results in more efficient DC activation and antigen presentation.

In the in vivo vaccine efficacy study, we observed that a low dose of cyclophosphamide (50 mg/kg) specifically depleted the regulatory T cell population (Figure 8). Berd and Mastrangelo reported that cyclophosphamide does not affect the CD4+CD25-T cell and CD8+ T cell populations [42,43,44]. The mechanism of action of cyclophosphamide against regulatory T cells is still unclear; however, Zhao et al. report that cyclophosphamide may act by reducing ATP levels in regulatory T cells. Low levels of ATP result in attenuation of glutathione synthesis within the cell, which leads to incomplete detoxification of cyclophosphamide [45]. In this study, we administered a low dose of cyclophosphamide (50 mg/kg) three days prior to administration of vaccine microparticles. Numerous reports have shown that a single dose, or continuous administration of low doses, of cyclophosphamide elicits long-term depletion of the regulatory T cell population. Sharabi et al. report that following cyclophosphamide dosing, the recovery of regulatory T cells takes more than 45 days [46,47,48]. Administration of a low dose of cyclophosphamide at regular intervals limits the cytotoxic effect on normal cells. In this study, we found that low doses of cyclophosphamide selectively deplete regulatory T cells with no effect on CD8+ T cells (Figure 9). An elevation in the CD8+ T cell population was observed in mice that received cyclophosphamide along with orally administered vaccine microparticles (Figure 9). Cyclophosphamide significantly depleted the central memory T cell population (Figure 10). No significant difference was observed between the groups with respect to the overall CD4+ T cell population (data not shown). In addition, animals that did not receive cyclophosphamide exhibited similar CD4+ T cell levels. These findings confirm that cyclophosphamide exerts minimal or no effects on the population of other T cells. Elevated CD8+ T cell populations in animals receiving combination therapy resulted in significant reduction in tumor growth compared with animals receiving single or no treatment (Figure 6). In order to elucidate the effects of cyclophosphamide on tumor regression, we administered cyclophosphamide via the intraperitoneal route. Significantly low tumor volumes were observed in mice that received only a low dose of cyclophosphamide; however, following termination of cyclophosphamide dosing, a gradual increase in tumor volume occurred, suggesting that lone treatment approach with cyclophosphamide is not expected to be effective and that adjunct treatment strategies would be required for effective inhibition of tumor growth.

The results of this study indicate that administering a low dose of cyclophosphamide can effectively decrease the population of regulatory T cells in the spleen. However, the tumor microenvironment (TME) plays a crucial role in both cancer progression and treatment. The TME consists of a variety of cell types, including fibroblasts, endothelial cells, immunosuppressive cells such as regulatory T cells, and other suppressor cells that can hinder immunity through the release of cytokines. Recent research has shown that cyclophosphamide can deplete Tregs and upregulate CD4+ and CD8+ cells in the TME [49]. In a future study, it would be interesting to investigate the effects of administering cancer vaccine microparticles and low dose cyclophosphamide simultaneously on immune cells within the TME.

## 5. Conclusions

In this study, mice receiving oral breast cancer vaccine microparticles administered with a low dose of cyclophosphamide exhibited a strong CD8+ T cell response against TAAs, that led to tumor regression. There was no significant difference in the CD4+ T cell population between groups receiving the vaccine with or without cyclophosphamide. This study demonstrates that combining vaccine therapy with low doses of cyclophosphamide induced a specific CD8+ T cell-mediated response. The present combinatorial approach offers a novel treatment strategy for controlling cancer progression.

## Figures and Tables

**Table 1:** In vivo vaccination study: group distribution and dose regimen.

Group Name	Description	Treatment Administered			
		Day 0	Day 4	Day 6	Day 9 (2nd Dose)
Naïve	No treatment	Tumor inoculation			0
Orally administered blank (OB)	Blank microparticles + No Cy	Tumor inoculation	Blank microparticles		Blank microparticles
Orally administered vaccine microparticles (OV)	Orally administered vaccine microparticles (10 mg/dose)	Tumor inoculation	Orally administered vaccine microparticles		Orally administered vaccine microparticles
Orally administered vaccine microparticles + Cyclophosphamide (OCy)	Orally administered vaccine microparticles (10 mg/dose) + Cyclophosphamide (50 mg/kg) (i.p.)	Tumor inoculation	Orally administered vaccine microparticles	50 mg/kg Cy (i.p.)	Orally administered vaccine microparticles
Cyclophosphamide only (Cy)	Cyclophosphamide (50 mg/kg) (i.p.)	Tumor inoculation	-	50 mg/kg Cy (i.p.)	-

## Data Availability

Data will be made available upon reasonable request.
